# Preparation and regeneration of iron-modified nanofibres for low-concentration phosphorus-containing wastewater treatment

**DOI:** 10.1098/rsos.190764

**Published:** 2019-09-04

**Authors:** Ying Luo, Min Liu, Ying Chen, Tingting Wang, Wei Zhang

**Affiliations:** 1 College of Architecture and Environment, Sichuan University, Chengdu 610065, People's Republic of China; 2 Sino-German Centre for Water and Health Research, Chengdu 610065, People's Republic of China; 3 State Key Laboratory of Polymer Materials Engineering, Polymer Research Institute at Sichuan University, Chengdu 610065, People's Republic of China

**Keywords:** ferric hydroxide, regeneration, bamboo nanocellulose, adsorption

## Abstract

In this study, nanocellulose (CNFs) was prepared by a mechanical shearing method, a simple and pollution-free process. Iron hydroxide was loaded on nanocellulose, a natural macromolecule derived from bamboo, to produce the second-generation iron-loaded nanocellulose for the removal of low-concentration phosphorus from wastewater. We found that the best modified ferric salt was ferric chloride. When the mass ratio of Fe(OH)_3_ and CNFs was 1.5 : 1, freeze-drying with liquid nitrogen yielded the best adsorption performance. The adsorption process of Fe(OH)_3_@CNFs followed the pseudo-second-order kinetics and belonged to chemical adsorption. Regeneration experiments showed that after 10 cycles of adsorption–regenerations of the adsorbent, the phosphorus adsorption efficiency was still stable at 80% of the initial material. The prepared adsorbent was characterized by the BET surface area measurement, scanning electron microscopy and FT-IR. The surface morphology, pore size and elements of materials before and after iron loading were analysed. Compared with other adsorbents, the phosphorus removal performances of the second-generation iron-loaded nanocellulose were superior. Compared with the first-generation material, the second-generation adsorbent is simpler and more environmentally friendly.

## 1. Introduction

Water pollution such as eutrophication is a worldwide problem [1]. Controlling phosphorus levels is an important means to abate water eutrophication and improve the water environment. China's ‘Discharge standard of pollutants for municipal wastewater treatment plant’ (GB18918-2002) stipulates that the first-class A standard of total phosphorus emission concentration is 0.5 mg l^−1^. In recent years, some local standards have been promulgated to make the total phosphorus emission standards more stringent, even down to 0.3 mg l^−1^, reaching the surface water category IV standard of China. Thus, there is an urgent need to develop efficient and stable technologies to remove low-concentration phosphorus from wastewater.

Adsorption is commonly used for treating low-concentration pollutants [2,3], due to the facile operation and low energy consumption [4–6]. In the removal of phosphorus, many natural adsorbents are developed, including fly ash [7–9], zeolite [10–14] and diatomite [15,16]. Fly ash has a strong adsorption capacity but may pollute the environment [17]. Zeolites have a large specific surface area and strong electrostatic attraction. However, natural zeolite exhibits a poor performance in removing anions directly from water. Diatomite has a large specific surface area and strong adsorption capacity for many pollutants. But due to the negative surface charges, diatomite is often used to adsorb positively charged heavy metal ions.

Cellulose is the most abundant and reproducible natural macromolecular material on Earth, and it possesses some adsorptive properties [18] and can be used as an adsorbent [19]. Bamboo is a cellulose-rich material with a high cellulose content [20]. China has a large bamboo production, and bamboo grows fast. The bamboo resources can be quickly regenerated. It is a high-quality cellulose material. In recent years, due to the high surface area of nanomaterials, researchers have been working on the preparation of nanomaterials as nanosorbents [21–29]. And there are few studies on bamboo-based cellulose at the nanoscale. Therefore, it is valuable to study the application of bamboo nanofibres adsorbent.

Natural bamboo materials have a weak adsorption capacity. It is generally necessary to modify the material to improve its adsorption performance. A large number of studies have shown that metal oxides and metal hydroxides, such as those of Fe [30–32], Al [33,34], Mn [35], Mg [2] and Zr [36,37], have a good adsorption activity on phosphorus. Among them, Fe and Al are the two most studied metals. Rebosura *et al*. [38] have shown that iron salts are commonly used in wastewater treatment and can also remove phosphorus from wastewater. At present, the development of highly efficient, environmentally friendly and repeatedly usable adsorbents is a research hotspot for phosphorus removal. In particular, the regeneration of adsorbent has attracted growing attention from researchers. Regeneration is the reverse process of adsorption. Reagent regeneration allows the recycling of the adsorbent, prolonging the life cycle of the adsorbent and reducing the cost of treatment.

Our research group has developed the first-generation iron-loaded nanocellulose (Fe(OH)_3_@CNFs) for phosphorus removal. Cui *et al*. [39] verified it can remove phosphorus from wastewater. But, cellulose produced by TEMPO is expensive and limits the mass production or applications. Therefore, in this study, the pristine bamboo pulp without drying or heating treatment was directly used as raw material to prepare nanocellulose using mechanical shearing. Iron hydroxide was loaded on this bamboo-based nanocellulose, developing the second-generation iron-loaded nanocellulose to improve the removal of the low-concentration phosphorus from wastewater. The effects of different iron salts, Fe(OH)_3_ and nanocellulose (CNFs) mass ratios, freezing concentration and freezing mode modification on the second-generation iron-loaded nanocellulose adsorption performance were studied. The regeneration properties of the material were also evaluated.

## 2. Material and methods

### 2.1. Chemical reagents

The chemical reagents mainly included: sodium hydroxide (NaOH), potassium phosphate monobasic (KH_2_PO_4_), sulfuric acid (H_2_SO_4_), hydrogen chloride (HCl), ascorbic acid (C_6_H_8_O_6_), ammonium molybdate ((NH_4_)_6_MO_7_O_4_·4H_2_O), potassium antimony(III)oxide tartrate hemi-hydrate (C_4_H_4_KO_7_Sb·1/2H_2_O), ferric nitrate (Fe(NO)_3_·9H_2_O), ferric chloride (FeCl_3_·6H_2_O), sodium chloride (NaCl), sodium nitrate (NaNO_3_), sodium sulfate (Na_2_SO_4_), ammonium chloride (NH_4_Cl), sodium carbonate (Na_2_CO_3_), potassium persulfate (K_2_S_2_O_8_), ferric sulfate (Fe_2_(SO_4_)_3_) and ferric reagent (LH-FE). Ferric sulfate was purchased from Xilong Chemical Co., Ltd, China, and LH-FE reagent was purchased from Beijing Lianhua Yongxing Technology Development Co., Ltd. The other reagents were purchased from Kelong Chemical Reagent Factory in Chengdu. All the chemicals were analytical grade. The simulated phosphorus-containing wastewater was prepared using potassium dihydrogen phosphate. The bamboo CNFs used in the experiment were provided by the State Key Laboratory of Polymer Materials Engineering, Polymer Research Institute at Sichuan University, China, in a 1 wt% solution.

### 2.2. Preparation of the second-generation iron-loaded nanocellulose and its phosphorus adsorption experiments

**Figure 1.**
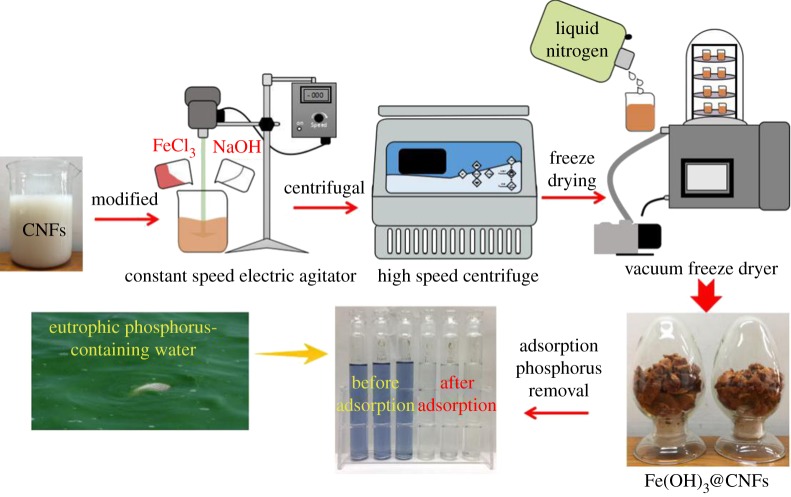
Schematic diagram of the optimized materials experimental programme.

The experimental procedure is shown in figure 1. The nanocellulose suspension was prepared by mechanical shearing of the pristine bamboo pulp as raw feedstock material [40]. The ferric chloride solution and sodium hydroxide solution were properly dosed into the CNF suspension. After stirring at 25°C for 10 h, the solution was washed by centrifugation, and the centrifuged solid was freeze-dried.

The experimental procedure consisted of adding 100 ml of the simulated wastewater containing 2 mg l^−1^ phosphate to 250 ml Erlenmeyer flask. After adding 10 mg adsorbent, the mixture was shaken in a constant temperature oscillation box at 25°C at 150 r.p.m. for 24 h. The suspension was filtered with a 0.45 µm polyethersulfone filter. Then the phosphorus content in the solution was measured before and after adsorption. When the impact of pH was explored, pH was adjusted to 4, 7 and 10. In other experiments, the pH was adjusted to 7. Three parallel experiments were performed for each group.

### 2.3. Regeneration experiments

The collected adsorbent was washed six times with deionized water and dried at 60°C for 24 h. In the sixth cycle of regeneration, the drying temperature of the adsorbent was raised to 105°C. The NaOH solution served as a desorption solution. The dried adsorbent was added to the desorption solution, shaken and immersed for 24 h in a 25°C constant temperature oscillator.

### 2.4. Analytical methods

The ammonium molybdate spectrophotometric method was employed to determine the phosphorus concentration in the solution. The iron concentration in the solution was measured using the bathophenanthroline spectrophotometric method. The mass concentration of blank nanofibres was determined by the dry weight method. Surface morphology and element content were analysed by a LEO 1530 scanning electron microscope (Zeiss, Germany). FT-IR analysis was performed using a Nicolet 6700 Fourier transform infrared spectrometer. The equilibrium adsorption capacity is calculated as shown in the following formula:

2.1 $$q_{e}=\frac{(C_{0}-C_{e})V}{m},$$

where *C*_0_ is the initial concentration (mg l^−1^), *C*_e_ is the concentration of the solution after adsorption (mg l^−1^), *q*_e_ is the adsorption capacity at adsorption equilibrium (mg g^−1^), *V* is the volume of desorption solution (l) and *m* is the mass of the adsorbent (g).

## 3. Results and discussions

### 3.1. Characterization

The amount of phosphorus adsorbed on this second-generation iron-loaded nanocellulose increased significantly compared with CNFs without the iron oxide modification. To better analyse the modified material, the material prepared under optimal conditions was characterized.

#### 3.1.1. SEM and EDS analyses

**Figure 2.**
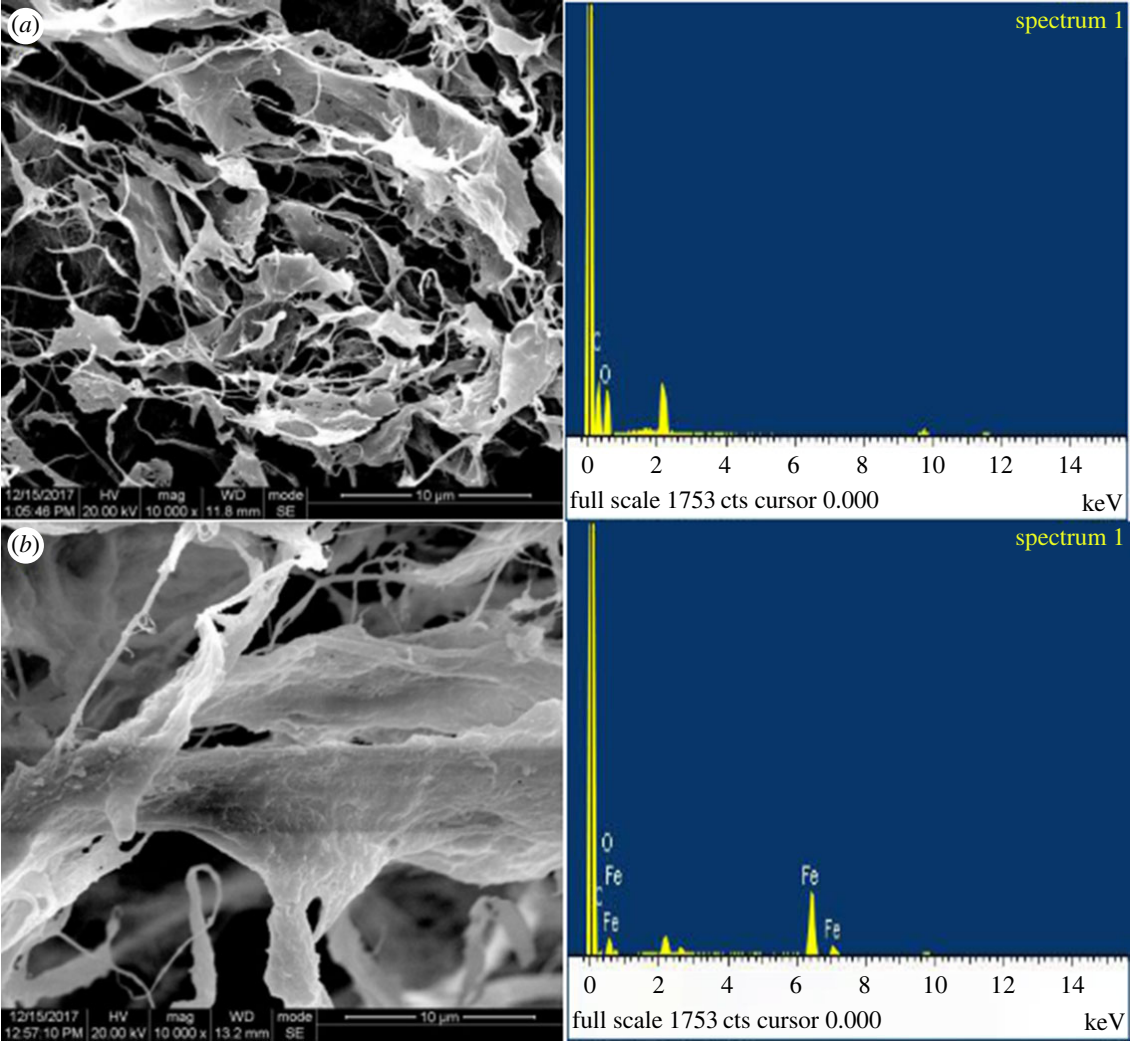
Scanning electron microscope (*a*) CNFs × 10 000 and (*b*) Fe(OH)_3_@CNFs × 10 000.

Figure 2 shows the fibrous structures of the pristine CNFs and the iron-loaded CNFs. The surface deposition of Fe(OH)_3_ on nanocellulose is confirmed by the EDS analysis. Table 1 shows that the surface-attached iron on CNFs reached a weight percentage of 33.65%.

**Table 1.** Analysis of CNFs elements before and after modification.

elements	CNFs (%)	Fe(OH)_3_@CNFs (%)
C	55.1	53.81
O	44.9	12.54
M	0	33.65

#### 3.1.2. Specific surface area and pore size analysis

Specific surface area, pore size distribution, pore size and pore volume are important for the adsorption performance of adsorbents. Table 2 shows that the specific surface area increased from 14.48 to 138.91 m^2^ g^−1^ after surface modification with Fe(OH)_3_. The specific surface area of the iron-loaded CNFs is about 9.6 times that of the pristine CNFs. The total pore volume reduced from 0.35 to 0.12 cm^3^ g^−1^ after the iron loading. The average pore diameter also reduced from 97.38 to 3.52 nm after modification. Fe(OH)_3_ is loaded on the surface of the material and inside the pores after the material modification, the particles of ferric hydroxide may increase the specific surface area of the material. The reduction in pore volume and pore diameter is presumably because of the surface deposition of Fe(OH)_3_.

**Table 2.** Analysis of specific surface area and pore size of CNFs before and after modification.

analyte	*S*_BET_ (m^2^ g^−1^)	Langmuir specific surface area (m^2^ g^−1^)	total pore volume (cm^3^ g^−1^)	average pore diameter (nm)	BJH average pore diameter (nm)
CNFs	14.481	19.412	0.353	97.379	200.540
Fe(OH)_3_@CNFs	138.907	189.214	0.122	3.518	2.052

#### 3.1.3. FT-IR analysis

**Figure 3.**
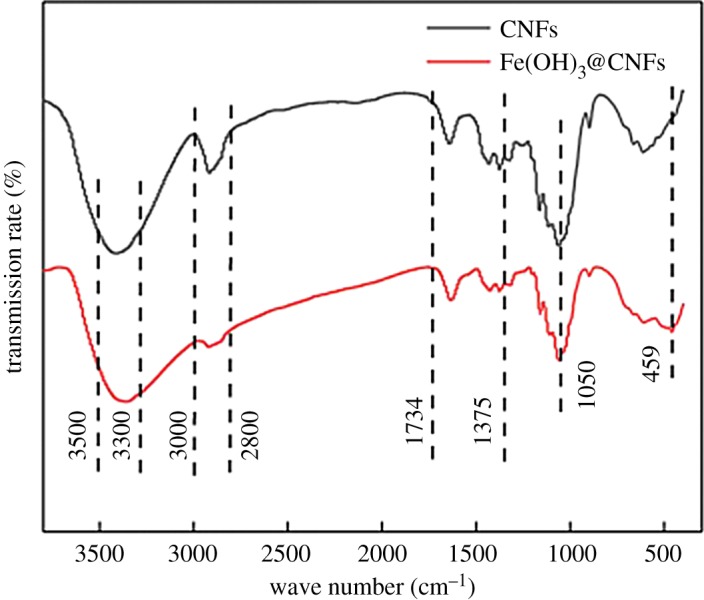
Infrared spectra of CNFs before and after modification.

Figure 3 compares the IR spectra of the pristine CNFs and the iron-loaded CNFs, which have similar patterns of surface functional groups. According to the relevant literature [41–43], the strong wide peaks between 3300 and 3500 cm^−1^ were –OH stretching vibration peaks. The peak near 2800–3000 cm^−1^ corresponded to the C–H symmetric stretching vibration absorption peak. The peak between 1375 and 1734 cm^−1^ and the peak near 899 cm^−1^ corresponded to the C–H deformation vibration absorption peak. The peak near 1050 cm^−1^ corresponded to C–O vibration. Compared with the pristine CNFs, the second-generation iron-loaded nanocellulose appeared to have a new peak at 459 cm^−1^, which could be ascribed to a functional group (Fe-OH) or the iron-hydroxyl bond.

### 3.2. Comparison of adsorption capacity of phosphorus

**Figure 4.**
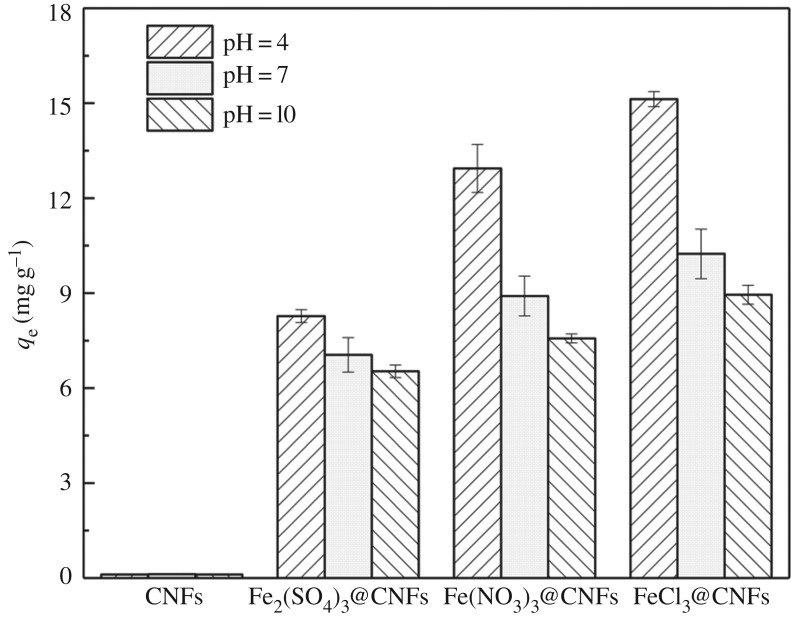
Phosphorus removal effect of CNFs and Fe(OH)_3_@CNFs under different pH conditions.

The adsorption efficiency of phosphorus on the pristine and the iron-loaded CNFs were studied at pH 4, 7 and 10. Three kinds of iron-loaded CNFs were prepared using ferric chloride, ferric sulfate and ferric nitrate. The modified CNFs had the same mass ratio of Fe(OH)_3_ and CNFs of 1 : 1. As shown in figure 4, the pristine CNFs had almost negligible phosphorus removal. By contrast, the phosphorus adsorption of the three modified CNFs was significantly improved and ferric chloride had the best phosphorus removal performance, but exhibited consistent pH dependence, which agrees with the previous study [44]. Under similar experimental conditions [45–48], the second-generation iron-loaded nanocellulose had better adsorption effect for phosphorus than most other adsorbents. The modification method is easy to operate. The phosphorus removal capacity of the optimized adsorbent was significantly enhanced.

### 3.3. Optimization of the preparation conditions for the second-generation iron-loaded nanocellulose

#### 3.3.1. Effect of the freezing concentration and freezing method

To investigate the effects of the second-generation iron-loaded nanocellulose prepared by varying the freezing concentrations and freezing methods on phosphorus removal, the prepared material solution was separately dispersed in water to 0.1, 1, 3, 5 and 10 wt%. The solutions were frozen with liquid nitrogen and a refrigerator. After freezing, the solutions were dried in a freeze dryer.

**Figure 5.**
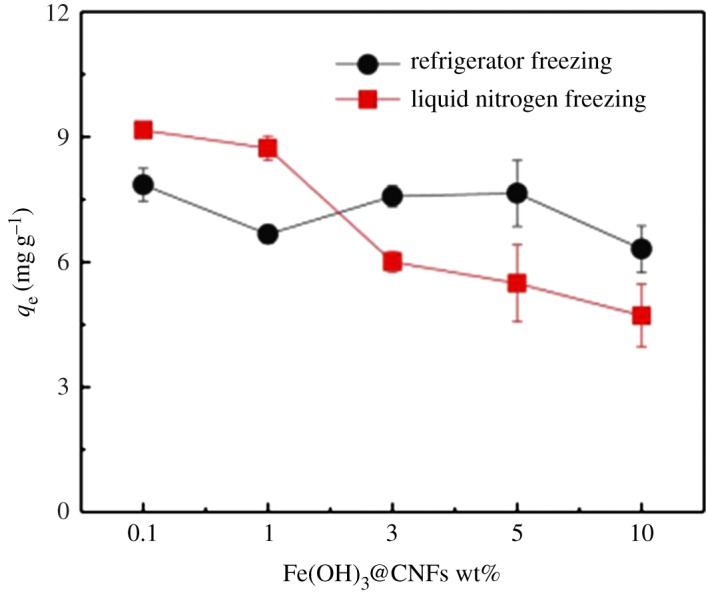
Phosphorus removal effect of different concentrations of Fe(OH)_3_@CNFs after freezing in refrigerator and liquid nitrogen.

According to figure 5, the adsorption capacity of the adsorbent slightly decreased after the freezing treatment by liquid nitrogen when the concentrations of the iron-loaded nanocellulose increased from 0.1 to 10 wt%. However, under the refrigerator freezing treatment, the adsorption capacity remained almost unchanged. The difference in the adsorption capacity after liquid nitrogen freeze-drying or freeze-drying in the refrigerator may be caused by the morphology changes of adsorbents during freezing/drying processes. Liquid nitrogen freezes the adsorbents quickly, and the water in the adsorbent solution can quickly become small, dense ice crystals. After vacuum drying, as the water is removed or vaporized, the adsorbent developed a porous structure with high specific surface area [49,50]. By contrast, the freezing time in the refrigerator is longer; accordingly, the ice crystals formation took a longer time during the freezing process and the ice crystals formed are larger. Therefore, liquid nitrogen freeze-dried materials were superior to refrigerator freeze-drying. At high mass concentrations, the freezing time of the adsorbents may be longer, which reduces the formation of the porous structures of the adsorbents. Unless indicated, the iron-loaded adsorbents were all treated under liquid nitrogen freezing at 1 wt%.

#### 3.3.2. Effect of the mass ratio of Fe(OH)_3_ and CNFs

**Figure 6.**
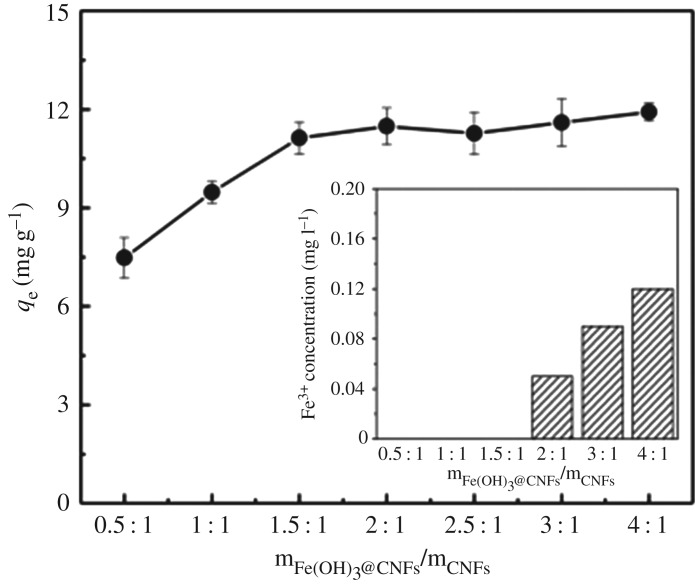
Phosphorus removal effect of the second-generation iron-loaded nanocellulose. Modified by different Fe(OH)_3_ and CNFs mass ratio. The inset shows iron dissolution.

To determine the optimal fabrication condition, the CNFs were modified by varying the mass ratio of Fe(OH)_3_/CNFs from 0.5 : 1 to 4 : 1 and the adsorption capacities were evaluated. Figure 6 shows that when the Fe(OH)_3_/CNF ratio was 0.5 : 1 ∼ 1.5 : 1, the adsorption capacity almost linearly increased with the mass ratio. When the Fe(OH)_3_/CNF ratio was 2 : 1 ∼ 4 : 1, the increase in adsorption capacity became less significant, indicating that the maximum active adsorption sites on iron-loaded CNFs could be reached.

Before the mass ratio of Fe(OH)_3_/CNFs was 1.5 : 1, no iron ions overflowed in the solution. With a 2 : 1 ratio, iron ions overflowed. The iron ions concentration increased as the mass ratio increased. CNFs could fully bind to Fe(OH)_3_ when the mass ratio of Fe(OH)_3_/CNFs was 1.5 : 1. With the increase in the Fe(OH)_3_, some Fe(OH)_3_ was not tightly bound to CNFs, which could be easily shaken off during the oscillating adsorption process. According to the phosphorus adsorption capacity and the amount of iron ion overflowed, and the mass ratio of Fe(OH)_3_/CNFs for modification was 1.5 : 1.

After optimization, the second-generation iron-loaded nanocellulose is used to adsorb phosphorus solution with an initial concentration of 2 mg l^−1^, and the adsorption capacity is 11.45 mg g^−1^. Cui *et al*. [39] developed the first-generation Fe(OH)_3_@CNFs. Cui *et al.* used Fe(OH)_3_@CNFs to adsorb phosphorus, and the maximum adsorption capacity was 142.86 mg g^−1^. The data in this study is far lower than that of Cui *et al*., mainly because of the different initial phosphorus concentrations (2 vs 500 mg l^−1^). However, when tested under the same condition (500 mg l^−1^), the maximum adsorption capacity of the present iron-loaded nanocellulose is 152.11 mg g^−1^, which is superior to that of Cui *et al.*

### 3.4. Adsorption kinetics

**Figure 7.**
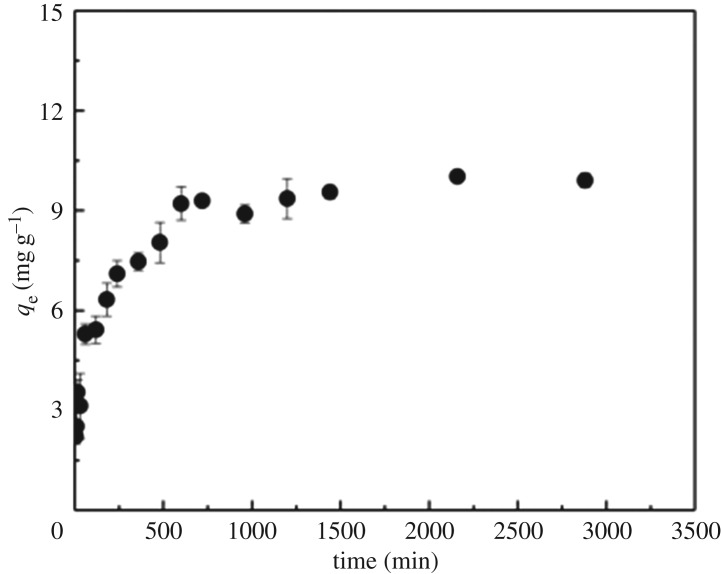
Sorption kinetics of phosphate on Fe(OH)_3_@CNFs.

The adsorption kinetics of phosphate on Fe(OH)_3_@CNFs increased rapidly in the first 11 h, and then reached equilibrium as shown in figure 7. The pseudo-first-order, the pseudo-second-order and Morris–Weber internal diffusion models were used to fit the adsorption kinetic data with the major fitting parameters shown in table 3. The pseudo-second-order kinetic model yielded a higher correlation coefficient (*R*^2^) of 0.998 than the pseudo-first-order kinetic model, suggesting that the adsorption process could be chemical adsorption. After fitting the kinetic data with Morris–Weber intraparticle diffusion model, indicating that internal diffusion was not the only step to control the adsorption process. The adsorption process was divided into two processes: surface adsorption and internal diffusion.

**Table 3.** Parameters for the adsorption kinetics model of Fe(OH)_3_@CNFs.

	pseudo-first-order model			pseudo-second-order model			Morris–Weber intraparticle diffusion model	
adsorbent	*q*_e_	*k*_1_	*R*^2^	*q*_e_	*k*_2_	*R*^2^	*k*_int_	*R*^2^
	mg g^−1^	min^−1^		mg g^−1^	g (mg min)^−1^		g (mg min^1/2^)^−1^	
Fe(OH)_3_@ CNFs	8.95	0.011	0.809	10.14	0.001	0.998	0.158	0.816

### 3.5. Regeneration studies

#### 3.5.1. Effect of NaOH solution concentration

**Figure 8.**
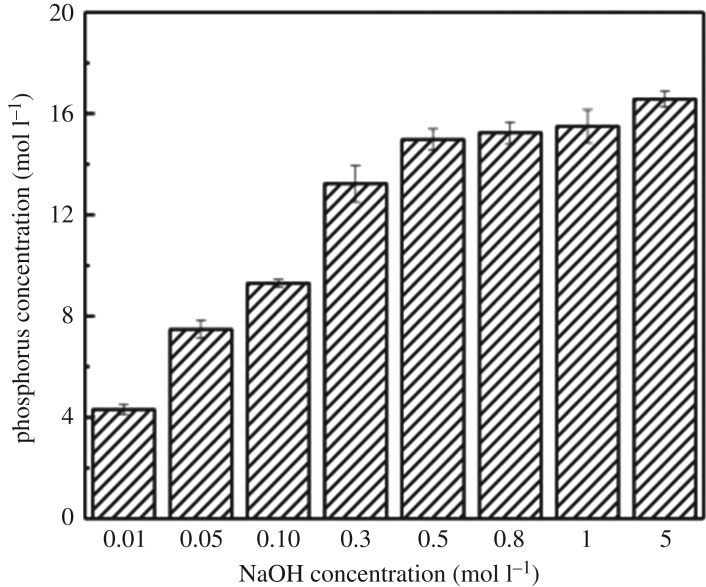
Effect of NaOH concentration on desorption.

Studies have shown that the sodium hydroxide (NaOH) cleaning is effective for phosphorus desorption and adsorbent regeneration and can be re-used many times after regeneration [51,52]. The NaOH solutions with different concentrations of 0.01–5 mol l^−1^ were used to remove phosphate from used adsorbents. Figure 8 shows that with the increase in NaOH concentration, the desorbed amounts of phosphorus from the adsorbent also increased and reached a plateau at an NaOH concentration of 0.5 mol l^−1^. Therefore, 0.5 mol l^−1^ NaOH solution was selected as the desorption solution.

#### 3.5.2. Effect of the regeneration times on the adsorption–desorption of phosphorus on the second-generation iron-loaded nanocellulose

**Figure 9.**
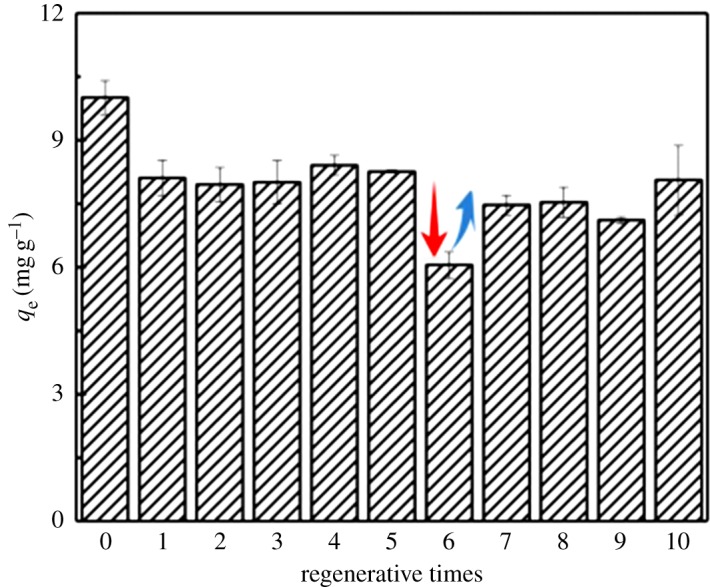
Effect of regeneration times on adsorption efficiency of the second-generation iron-modified nanocellulose.

After 10 cycles of adsorption and desorption using the above regeneration process, the adsorption capacity slightly decreased by 19% compared to the initial adsorption capacity as shown in figure 9. In the sixth cycle of regeneration, the drying temperature of the adsorbent was raised to 105°C, which led to the decrease in phosphorus adsorption. In the seventh cycle of regeneration, the drying temperature was reduced to 60°C, which increased the adsorption capacity to more than 80% of the initial level. Clearly, the high drying temperature caused the decline of adsorption affinity probably because of the reduction in adsorption sites. However, lowering the drying temperature could recover the adsorption capacity of the iron-loaded CNFs.

## 4. Conclusion

In this study, nanocellulose was produced using mechanical shearing of wet bamboo pulp as raw material. This method does not use TEMPO reagent, which simplifies the synthesis procedure and reduces secondary pollution from adsorbent production. After optimizing the preparation conditions of the iron-loaded nanocellulose, ferric chloride was determined to be the best modifier chemical. The specific surface area of the modified adsorbent increased from 14.48 to 138.91 m^2^ g^−1^. When the mass ratio of Fe(OH)_3_ to CNFs was 1.5 : 1, the addition of FeCl_3_ solution and freeze-drying with liquid nitrogen yielded the highest adsorption capacity on phosphorus. The adsorption process of Fe(OH)_3_@CNFs conforms to the pseudo-second-order kinetic model, suggesting that the adsorption process is chemical adsorption. After 10 desorption–adsorption regeneration cycles, the phosphorus adsorption capacity remained stable. The second-generation iron-loaded nanocellulose can be re-used at least 10 times. This iron-loaded nanocellulose holds great promise as highly effective and economically viable phosphorus adsorbent.

## Supplementary Material

Reviewer comments

## Data accessibility

Data available from the Dryad Digital Repository: https://doi.org/10.5061/dryad.c53m9d5 [53].

## References

[1] Abdelrahman EA, Hegazey RM 2019 Facile synthesis of HgO nanoparticles using hydrothermal method for efficient photocatalytic degradation of crystal violet dye under UV and sunlight irradiation. J. Inorg. Organomet. Polym. 29, 346–358. (10.1007/s10904-018-1005-6)

[2] Han C, Lalley J, Iyanna N, Nadagouda M 2017 Removal of phosphate using calcium and magnesium-modified iron-based adsorbents. Mater. Chem. Phys. 198, 115–124. (10.1016/j.matchemphys.2017.05.038)

[3] Abdelrahman EA, Tolan DA, Nassar MY 2019 A tunable template-assisted hydrothermal synthesis of hydroxysodalite zeolite nanoparticles using various aliphatic organic acids for the removal of zinc(II) ions from aqueous media. J. Inorg. Organomet. Polym. 29, 229–247. (10.1007/s10904-018-0982-9)

[4] Wang X, Jiang C, Hou B, Wang Y, Chen H, Wu J 2014 Carbon composite lignin-based adsorbents for the adsorption of dyes. Chemosphere 206, 587–596. (10.1016/j.chemosphere.2018.04.183) 29778084

[5] Xiong L, Longqi X, Yaqing L, Wenqi Z 2018 Synthesis of citric acid-modified resins and their adsorption properties towards metal ions. R. Soc. open sci. 5, 8 (10.1098/rsos.171667) PMC6124113 30224979

[6] Abdelrahman EA, Hegazey RM 2019 Utilization of waste aluminum cans in the fabrication of hydroxysodalite nanoparticles and their chitosan biopolymer composites for the removal of Ni(II) and Pb(II) ions from aqueous solutions: kinetic, equilibrium, and reusability studies. Microchem. J. 145, 18–25. (10.1016/j.microc.2018.10.016)

[7] Guan Q, Hu X, Wu D, Shang X, Ye C, Kong H 2009 Phosphate removal in marine electrolytes by zeolite synthesized from coal fly ash. Fuel 88, 1643–1649. (10.1016/j.fuel.2009.02.031)

[8] Xu K, Deng T, Liu J, Peng W 2010 Study on the phosphate removal from aqueous solution using modified fly ash. Fuel 89, 3668–3674. (10.1016/j.fuel.2010.07.034)

[9] Sima TV, Letshwenyo MW 2018 Efficiency of waste clinker ash and iron oxide tailings for phosphorus removal from tertiary wastewater: batch studies. Environ. Technol. Innov. 11, 49–63. (10.1016/j.eti.2018.04.008)

[10] Hamdi N, Srasra E 2012 Removal of phosphate ions from aqueous solution using Tunisian clays minerals and synthetic zeolite. J. Environ. Sci. China 24, 617–623. (10.1016/S1001-0742(11)60791-2) 22894095

[11] Hazrati H, Jahanbakhshi N, Rostamizadeh M 2018 Fouling reduction in the membrane bioreactor using synthesized zeolite nano-adsorbents. J. Membrane Sci. 555, 455–462. (10.1016/j.memsci.2018.03.076)

[12] Abdelrahman EA 2018 Synthesis of zeolite nanostructures from waste aluminum cans for efficient removal of malachite green dye from aqueous media. J. Mol. Liq. 253, 72–82. (10.1016/j.molliq.2018.01.038)

[13] Nassar MY, Abdelrahman EA 2017 Hydrothermal tuning of the morphology and crystallite size of zeolite nanostructures for simultaneous adsorption and photocatalytic degradation of methylene blue dye. J. Mol. Liq. 242, 364–374. (10.1016/j.molliq.2017.07.033)

[14] Aly HM, Moustafa ME, Abdelrahman EA 2012 Synthesis of mordenite zeolite in absence of organic template. Adv. Powder Technol. 23, 757–760. (10.1016/j.apt.2011.10.003)

[15] Xie F, Wu F, Liu G, Mu Y, Feng C, Wang H, Giesy JP 2014 Removal of phosphate from eutrophic lakes through adsorption by *in situ* formation of magnesium hydroxide from diatomite. Environ. Sci. Technol. 48, 582–590. (10.1021/es4037379) 24328241

[16] Xiong W, Peng J 2008 Development and characterization of ferrihydrite-modified diatomite as a phosphorus adsorbent. Water Res. 42, 4869–4877. (10.1016/j.watres.2008.09.030) 18947855

[17] Wang Z, Fan Y, Li Y, Qu F, Wu D, Kong H 2016 Synthesis of zeolite/hydrous lanthanum oxide composite from coal fly ash for efficient phosphate removal from lake water. Microporous Mesoporous Mater. 222, 226–234. (10.1016/j.micromeso.2015.10.028)

[18] Liu P, Borrell PF, Božič M, Kokol V, Oksman K 2015 Nanocelluloses and their phosphorylated derivatives for selective adsorption of Ag^+^, Cu^2+^ and Fe^3+^ from industrial effluents. J. Hazard. Mater. 294, 177–185. (10.1016/j.jhazmat.2015.04.001) 25867590

[19] Ma Z, Li Q, Yue Q, Gao B, Li W, Xu X, Zhong Q 2011 Adsorption removal of ammonium and phosphate from water by fertilizer controlled release agent prepared from wheat straw. Chem. Eng. J. 171, 1209–1217. (10.1016/j.cej.2011.05.027)

[20] Wang H, Zhang X, Jiang Z, Li W, Yu Y 2015 A comparison study on the preparation of nanocellulose fibrils from fibers and parenchymal cells in bamboo (*Phyllostachys pubescens*). Ind. Crops Prod. 71, 80–88. (10.1016/j.indcrop.2015.03.086)

[21] Abdelrahman EA, Hegazey RM 2019 Exploitation of Egyptian insecticide cans in the fabrication of Si/Fe nanostructures and their chitosan polymer composites for the removal of Ni(II), Cu(II), and Zn(II) ions from aqueous solutions. Compos. Part B 166, 382–400. (10.1016/j.compositesb.2019.02.027)

[22] Nassar MY, Abdelrahman EA, Aly AA, Mohamed TY 2017 A facile synthesis of mordenite zeolite nanostructures for efficient bleaching of crude soybean oil and removal of methylene blue dye from aqueous media. J. Mol. Liq. 248, 302–313. (10.1016/j.molliq.2017.10.061)

[23] Nassar MY, Aly HM, Abdelrahman EA, Moustafa ME 2017 Synthesis, characterization, and biological activity of some novel Schiff bases and their Co(II) and Ni(II) complexes: a new route for Co_3_O_4_ and NiO nanoparticles for photocatalytic degradation of methylene blue dye. J. Mol. Struct. 1143, 462–471. (10.1016/j.molstruc.2017.04.118)

[24] Aly HM, Moustafa ME, Nassar MY, Abdelrahman EA 2015 Synthesis and characterization of novel Cu(II) complexes with 3-substituted-4-amino-5-mercapto-1,2,4-triazole Schiff bases: a new route to CuO nanoparticles. J. Mol. Struct. 1086, 223–231. (10.1016/j.molstruc.2015.01.017)

[25] Hanifehpour Y, Soltani B, Reza Amani-Ghadim A, Hodayi H, Min B, Woo Joo S 2017 Novel visible light photocatalyst based on holmium-doped cadmium sulfide: synthesis, characterization and kinetics study. J. Inorg. Organomet. Polym. 27, 1–14. (10.1007/s10904-016-0432-5)

[26] Abdelrahman EA, Hegazey RM, Kotp YH, Alharbi A 2019 Facile synthesis of Fe_2_O_3_ nanoparticles from Egyptian insecticide cans for efficient photocatalytic degradation of methylene blue and crystal violet dyes. Spectrochim. Acta, Part A 222, 117195 (10.1016/j.saa.2019.117195) 31176151

[27] Raghava Reddy K, Karthik KV, Benaka Prasad SB, Soni SK, Jeong HM, Raghu AV 2016 Enhanced photocatalytic activity of nanostructured titanium dioxide/polyaniline hybrid photocatalysts. Polyhedron 120, 169–174. (10.1016/j.poly.2016.08.029)

[28] Basheer AA 2018 New generation nano-adsorbents for the removal of emerging contaminants in water. J. Mol. Liq. 261, 583–593. (10.1016/j.molliq.2018.04.021)

[29] Inamuddin, Ahamed MI, Asiri AM, Lichtfouse E 2019 Nanophotocatalysis and Environmental Applications, pp. 83–105. Cham, Switzerland: Springer Nature Switzerland.

[30] Du X, Han Q, Li J, Li H 2017 The behavior of phosphate adsorption and its reactions on the surfaces of Fe–Mn oxide adsorbent. J. Taiwan Inst. Chem. E 76, 167–175. (10.1016/j.jtice.2017.04.023)

[31] Choi J, Chung J, Lee W, Lim H-S, Kim J-O 2016 Recovery of phosphate by magnetic iron oxide particles and iron oxide nanotubes in water. Water Air Soil Pollution 227, 1–11. (10.1007/s11270-016-2781-7)

[32] Ajmal Z, Muhmood A, Usman M, Kizito S, Lu J, Dong R, Wu S 2018 Phosphate removal from aqueous solution using iron oxides: adsorption, desorption and regeneration characteristics. J. Colloid Interface Sci. 528, 145–155. (10.1016/j.jcis.2018.05.084) 29843062

[33] Genz A, Kornmüller A, Jekel M 2004 Advanced phosphorus removal from membrane filtrates by adsorption on activated aluminium oxide and granulated ferric hydroxide. Water Res. 38, 3523–3530. (10.1016/j.watres.2004.06.006) 15325178

[34] Mor S, Chhoden K, Negi P, Ravindra K 2017 Utilization of nano-alumina and activated charcoal for phosphate removal from wastewater. Environ. Nanotechnol. Monit. Manage. 7, 15–23. (10.1016/j.enmm.2016.11.006)

[35] Liu T, Wu K, Zeng L 2012 Removal of phosphorus by a composite metal oxide adsorbent derived from manganese ore tailings. J. Hazard. Mater. 217–218, 29–35. (10.1016/j.jhazmat.2012.01.019) 22464586

[36] Chitrakar R, Tezuka S, Sonoda A, Sakane K, Ooi K, Hirotsu T 2006 Selective adsorption of phosphate from seawater and wastewater by amorphous zirconium hydroxide. J. Colloid Interface Sci. 297, 426–433. (10.1016/j.jcis.2005.11.011) 16337645

[37] Su Y, Cui H, Li Q, Gao S, Shang JK 2013 Strong adsorption of phosphate by amorphous zirconium oxide nanoparticles. Water Res. 47, 5018–5026. (10.1016/j.watres.2013.05.044) 23850213

[38] Rebosura M Jr, Salehin S, Pikaar I, Sun X, Keller J, Sharma K, Yuan Z 2018 A comprehensive laboratory assessment of the effects of sewer-dosed iron salts on wastewater treatment processes. Water Res. 146, 109–117. (10.1016/j.watres.2018.09.021) 30241044

[39] Cui G, Liu M, Chen Y, Zhang W, Zhao J 2016 Synthesis of a ferric hydroxide-coated cellulose nanofiber hybrid for effective removal of phosphate from wastewater. Carbohydr. Polym. 154, 40–47. (10.1016/j.carbpol.2016.08.025) 27577894

[40] Xiaofang Z, Jiangqi Z, Xu H, Qingye L, Chenghong A, Tian X, Wei Z, Canhui L, Yulin D 2018 Mechanically robust and highly compressible electrochemical supercapacitors from nitrogen-doped carbon aerogels. Carbon 127, 236–244. (10.1016/j.carbon.2017.10.083)

[41] Nguyen TAH, Ngo HH, Guo WS, Zhang J, Liang S, Tung KL 2013 Feasibility of iron loaded ‘okara’ for biosorption of phosphorous in aqueous solutions. Bioresour. Technol. 150, 42–49. (10.1016/j.biortech.2013.09.133) 24144598

[42] Bowden LI, Jarvis AP, Younger PL, Johnson KL 2009 Phosphorus removal from waste waters using basic oxygen steel slag. Environ. Sci. Technol. 43, 2476–2481. (10.1021/es801626d) 19452904

[43] Hui B, Zhang Y, Ye L 2014 Preparation of PVA hydrogel beads and adsorption mechanism for advanced phosphate removal. Chem. Eng. J. 235, 207–214. (10.1016/j.cej.2013.09.045)

[44] Zhang XN, Lin XY, He Y, Chen Y, Zhou J, Luo XG 2018 Adsorption of phosphorus from slaughterhouse wastewater by carboxymethyl konjac glucomannan loaded with lanthanum. Int. J. Biol. Macromol. 119, 105–115. (10.1016/j.ijbiomac.2018.07.140) 30036618

[45] Jiang F, Hsieh Y-L 2014 Super water absorbing and shape memory nanocellulose aerogels from TEMPO-oxidized cellulose nanofibrils via cyclic freezing-thawing. J. Mater. Chem. A 2, 350–359. (10.1039/C3TA13629A)

[46] Zhang X, Wang Y, Zhao J, Xiao M, Zhang W, Li C 2016 Mechanically strong and thermally responsive cellulose nanofibers/poly(N-isopropylacrylamide) composite aerogels. ACS Sustain. Chem. Eng. 4, 4321–4327. (10.1021/acssuschemeng.6b00814)

[47] Hokkanen S, Repo E, Sillanpää M 2013 Removal of heavy metals from aqueous solutions by succinic anhydride modified mercerized nanocellulose. Chem. Eng. J. 223, 40–47. (10.1016/j.cej.2013.02.054)

[48] Wang Y, Zhang X, He X, Zhang W, Zhang X, Lu C 2014 *In situ* synthesis of MnO_2_ coated cellulose nanofibers hybrid for effective removal of methylene blue. Carbohyd. Polym. 110, 302–308. (10.1016/j.carbpol.2014.04.008) 24906760

[49] Rout PR, Bhunia P, Dash RR 2015 A mechanistic approach to evaluate the effectiveness of red soil as a natural adsorbent for phosphate removal from wastewater. Desalin. Water Treat. 54, 358–373. (10.1080/19443994.2014.881752)

[50] Yoon S-Y, Lee C-G, Park J-A, Kim J-H, Kim S-B, Lee S-H, Choi J-W 2014 Kinetic, equilibrium and thermodynamic studies for phosphate adsorption to magnetic iron oxide nanoparticles. Chem. Eng. J. 236, 341–347. (10.1016/j.cej.2013.09.053)

[51] Awual MR, Jyo A, Ihara T, Seko N, Tamada M, Lim KT 2011 Enhanced trace phosphate removal from water by zirconium(IV) loaded fibrous adsorbent. Water Res. 45, 4592–4600. (10.1016/j.watres.2011.06.009) 21724222

[52] Ashekuzzaman SM, Jiang J-Q 2014 Study on the sorption–desorption–regeneration performance of Ca-, Mg- and CaMg-based layered double hydroxides for removing phosphate from water. Chem. Eng. J. 246, 97–105. (10.1016/j.cej.2014.02.061)

[53] Luo Y, Liu M, Chen Y, Wang T, Zhang W. 2019 Data from: Preparation and regeneration of iron-modified nanofibres for low-concentration phosphorus-containing wastewater treatment Dryad Digital Repository. (10.5061/dryad.c53m9d5) PMC6774935 31598304

